# *N*-[Eth­yl(2-hy­droxy­eth­yl)carbamo­thio­yl]-2-methyl­benzamide

**DOI:** 10.1107/S1600536814008952

**Published:** 2014-04-26

**Authors:** Bohari M. Yamin, Sara Maira M. Hizam, Siti Fairus M. Yusoff, Siti Aishah Hasbullah

**Affiliations:** a Low Carbon Research Group, School of Chemical Sciences and Food Technology, Universiti Kebangsaan Malaysia, 43600 Bangi, Selangor, Malaysia; b School of Chemical Sciences and Food Technology, Universiti Kebangsaan Malaysia, 43600 Bangi, Selangor, Malaysia

## Abstract

The title compound, C_13_H_18_N_2_O_2_S, adopts a *cis* conformation between the methyl­benzoyl and thiono groups across their thio­urea C—N bond. However, the methyl­benzoyl group and N_2_CS thio­urea moiety are twisted by 15.03 (3)°. In the molecule there is an N—H⋯O hydrogen bond. In the crystal, mol­ecules are linked by O—H⋯O inter­actions, generating chains extending along the *c*-axis direction.

## Related literature   

For bond-length data, see: Allen *et al.* (1987 ▶). For related structures of thio­urea derivatives, see: Awang *et al.* (2013 ▶); Sapari *et al.* (2013 ▶). 
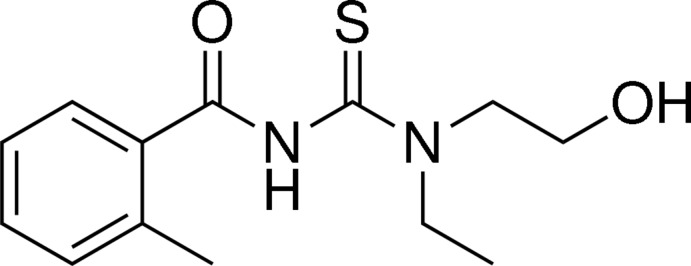


## Experimental   

### 

#### Crystal data  

C_13_H_18_N_2_O_2_S
*M* *_r_* = 266.35
Monoclinic, 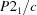

*a* = 11.393 (4) Å
*b* = 8.989 (3) Å
*c* = 14.467 (5) Å
β = 109.940 (9)°
*V* = 1392.7 (7) Å^3^
*Z* = 4
Mo *K*α radiation
μ = 0.23 mm^−1^
*T* = 296 K
0.35 × 0.34 × 0.06 mm

#### Data collection  

Bruker SMART APEX CCD area-detector diffractometer
Absorption correction: multi-scan (*SADABS*; Bruker, 2009 ▶) *T*_min_ = 0.924, *T*_max_ = 0.986
28196 measured reflections
2583 independent reflections
2005 reflections with *I* > 2σ(*I*)
*R*_int_ = 0.048

#### Refinement  

*R*[*F* ^2^ > 2σ(*F* ^2^)] = 0.036
*wR*(*F* ^2^) = 0.088
*S* = 1.06
2583 reflections
169 parameters
1 restraint
H atoms treated by a mixture of independent and constrained refinement
Δρ_max_ = 0.20 e Å^−3^
Δρ_min_ = −0.20 e Å^−3^

### 

Data collection: *SMART* (Bruker, 2009 ▶); cell refinement: *SAINT* (Bruker, 2009 ▶); data reduction: *SAINT*; program(s) used to solve structure: *SHELXS97* (Sheldrick, 2008 ▶); program(s) used to refine structure: *SHELXL97* (Sheldrick, 2008 ▶); molecular graphics: *SHELXTL* (Sheldrick, 2008 ▶); software used to prepare material for publication: *SHELXTL*, *PLATON* (Spek, 2009 ▶) and *publCIF* (Westrip, 2010 ▶).

## Supplementary Material

Crystal structure: contains datablock(s) global, I. DOI: 10.1107/S1600536814008952/lr2125sup1.cif

Structure factors: contains datablock(s) I. DOI: 10.1107/S1600536814008952/lr2125Isup2.hkl

Supporting information file. DOI: 10.1107/S1600536814008952/lr2125Isup3.cml

CCDC reference: 998473

Additional supporting information:  crystallographic information; 3D view; checkCIF report

## Figures and Tables

**Table 1:** Hydrogen-bond geometry (Å, °)

*D*—H⋯*A*	*D*—H	H⋯*A*	*D*⋯*A*	*D*—H⋯*A*
N1—H1*A*⋯O2	0.86	1.98	2.750 (2)	149
O2—H2*A*⋯O1^i^	0.81 (2)	1.91 (2)	2.716 (2)	171 (2)

Symmetry code: (i) 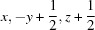.

**Fig. 1.**
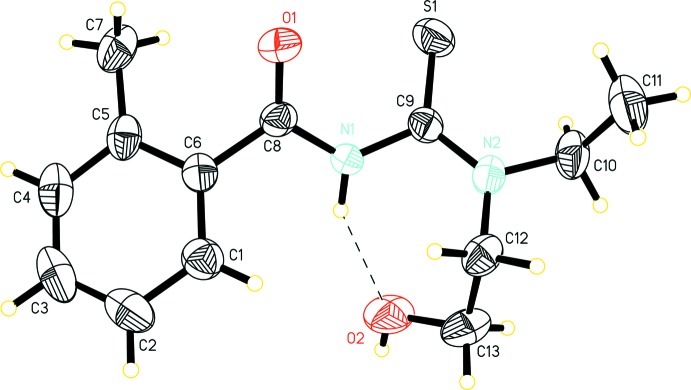
The molecular structure of (I), with displacement ellipsods drawn at the 50% probability level. The dashed line indicates the intramolecular hydrogen bond.

**Fig. 2.**
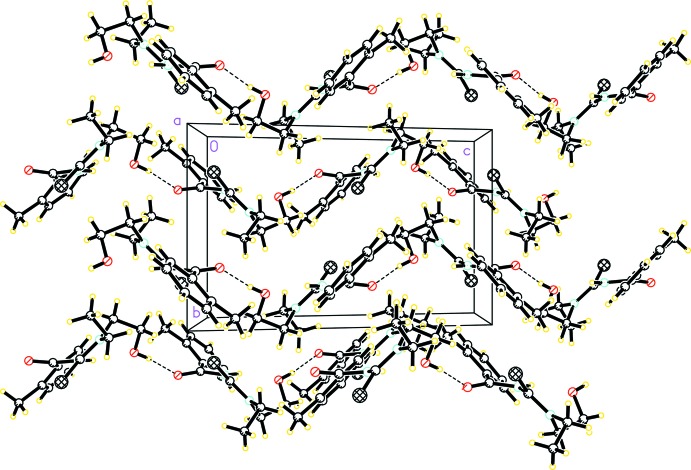
Packing of (I) viewed down the a-axis. The dashed lines indicate intermolecular hydrogen bonds.

## supplementary crystallographic information

### 2.1. Synthesis and crystallization

An acetone (30 ml) solution of 2-(ethyl­amino)­ethanol (0.18 g, 2 mmol) was added to a round-bottomed flask containing 2-methyl­benzoyl iso­thio­cyanate (0.31 g,2 mmol). The mixture was refluxed for 3h. After cooling the solution was filtered off and the filtrate was left to evaporate at room temperature. The solid formed was washed with water and cold ethanol. Crystals suitable for X-ray study were obtained by recrystallization from DMSO.

### 2.2. Refinement

Crystal data, data collection and structure refinement details are summarized in Table 1. All H atoms attached to C and N atoms were fixed geometrically and treated as riding with C—H= 0.93-0.97Å and N–H = 0.86Å with U_iso_(H)= 1.2U_eq_[C (methyl­ene and aromatic),N] and 1.5 U_eq_ [C (methyl)]. The hydroxyl hydrogen atom was located from Fourier map and refined isotropically with O—H restraint to 0.81Å with esd of 0.01.

### 3. Results and discussion

The carbonoyl­thio­urea derivatives with a secondary amine group at the terminal thio­urea moiety are expected to adopt a *cis* conformation with respect to the position of the carbonoyl against the thiono group. Such a configuration will allow the ligand to chelate with metals in bidentate manner. Thus, 2,4-di­chloro-N-[ethyl­(hy­droxy­ethyl)­carbamo­thioyl]benzamide (Sapari *et al.*,2013) and N[ethyl­(hy­droxy­ethyl)­carbamo­thioyl]-2-iodo-benzamide (Awang *et al.*, 2013) adopt the said conformation. The title compound is analogous to the two compounds but having a methyl group attached at position-2 of the benzene ring (Fig.1). However, the title molecule maintains a *cis* conformation between the carbonyl and thiono groups across the C8—N1 bond and twisted by torsion angle of O1—C8—N1—C9 and S1—C9—N1—C8 of 7.1 (3)and 47.1 (2)° respectively. Both S1/N1/N2/C9 thio­urea moiety and (C1—C8) benzyl fragments are planar with maximum deviation of 0.031 (2)Å for C4 atom from the least square plane of the benzyl fragment. The two planes make dihedral angle of 15.03 (3)°. The bond lengths and angles are in normal ranges (Allen *et al.*,1987) and comparable to those in the two analogs. There is an intra­molecular hydrogen bond N1–H1A···O2 between the amido hydrogen and hydroxyl oxygen atom. In the crystal structure, the molecules are linked by O2–H2A···O1 inter­molecular hydrogen bond (see Table 1 for symmetry codes) to form one-dimensional chains along the *c*-axis direction (Fig.2). In addition, there is a C—H.. π bond between H12B and (C1—C6) centroid (-x,1-y,-z) with the H12B···C_g_ distance of 2.84Å and C12—H12B—C_g_ angle, 137°.

### Crystal data

**Table:**

C_13_H_18_N_2_O_2_S	*F*(000) = 568
*M**_r_* = 266.35	*D*_x_ = 1.270 Mg m^−^^3^
Monoclinic, *P*2_1_/*c*	Mo *K*α radiation, λ = 0.71073 Å
Hall symbol: -P 2ybc	Cell parameters from 21535 reflections
*a* = 11.393 (4) Å	θ = 2.8–26.5°
*b* = 8.989 (3) Å	µ = 0.23 mm^−^^1^
*c* = 14.467 (5) Å	*T* = 296 K
β = 109.940 (9)°	Block, colorless
*V* = 1392.7 (7) Å^3^	0.35 × 0.34 × 0.06 mm
*Z* = 4	

### Data collection

**Table:**

Bruker SMART APEX CCD area-detector diffractometer	2583 independent reflections
Radiation source: fine-focus sealed tube	2005 reflections with *I* > 2σ(*I*)
Graphite monochromator	*R*_int_ = 0.048
Detector resolution: 83.66 pixels mm^-1^	θ_max_ = 25.5°, θ_min_ = 3.6°
ω scans	*h* = −13→13
Absorption correction: multi-scan (*SADABS*; Bruker, 2009)	*k* = −10→10
*T*_min_ = 0.924, *T*_max_ = 0.986	*l* = −17→17
28196 measured reflections	

### Refinement

**Table:**

Refinement on *F*^2^	Secondary atom site location: difference Fourier map
Least-squares matrix: full	Hydrogen site location: inferred from neighbouring sites
*R*[*F*^2^ > 2σ(*F*^2^)] = 0.036	H atoms treated by a mixture of independent and constrained refinement
*wR*(*F*^2^) = 0.088	*w* = 1/[σ^2^(*F*_o_^2^) + (0.0285*P*)^2^ + 0.6558*P*] where *P* = (*F*_o_^2^ + 2*F*_c_^2^)/3
*S* = 1.06	(Δ/σ)_max_ < 0.001
2583 reflections	Δρ_max_ = 0.20 e Å^−^^3^
169 parameters	Δρ_min_ = −0.20 e Å^−^^3^
1 restraint	Extinction correction: *SHELXL97* (Sheldrick, 2008), Fc^*^=kFc[1+0.001xFc^2^λ^3^/sin(2θ)]^-1/4^
Primary atom site location: structure-invariant direct methods	Extinction coefficient: 0.0087 (13)

### Special details

**Table:**

Geometry. All esds (except the esd in the dihedral angle between two l.s. planes) are estimated using the full covariance matrix. The cell esds are taken into account individually in the estimation of esds in distances, angles and torsion angles; correlations between esds in cell parameters are only used when they are defined by crystal symmetry. An approximate (isotropic) treatment of cell esds is used for estimating esds involving l.s. planes.
Refinement. Refinement of F^2^ against ALL reflections. The weighted R-factor wR and goodness of fit S are based on F^2^, conventional R-factors R are based on F, with F set to zero for negative F^2^. The threshold expression of F^2^ > 2sigma(F^2^) is used only for calculating R-factors(gt) etc. and is not relevant to the choice of reflections for refinement. R-factors based on F^2^ are statistically about twice as large as those based on F, and R- factors based on ALL data will be even larger.

### Fractional atomic coordinates and isotropic or equivalent isotropic displacement parameters (Å^2^)

**Table:**

	*x*	*y*	*z*	*U*_iso_*/*U*_eq_	
S1	0.41046 (5)	0.21117 (6)	0.04784 (4)	0.05354 (18)	
O1	0.13506 (12)	0.29922 (15)	−0.09305 (9)	0.0484 (3)	
O2	0.19987 (15)	0.33465 (18)	0.26307 (10)	0.0605 (4)	
N1	0.19870 (12)	0.29892 (16)	0.07400 (10)	0.0349 (3)	
H1A	0.1715	0.2966	0.1226	0.042*	
N2	0.37657 (13)	0.41992 (16)	0.16698 (10)	0.0360 (3)	
C1	−0.06300 (17)	0.3318 (2)	0.04619 (13)	0.0440 (4)	
H1	−0.0132	0.4036	0.0876	0.053*	
C2	−0.18157 (19)	0.3051 (3)	0.04839 (16)	0.0563 (6)	
H2	−0.2126	0.3604	0.0893	0.068*	
C3	−0.25302 (18)	0.1954 (3)	−0.01084 (16)	0.0588 (6)	
H3	−0.3319	0.1741	−0.0086	0.071*	
C4	−0.20788 (17)	0.1171 (2)	−0.07337 (15)	0.0516 (5)	
H4	−0.2572	0.0427	−0.1123	0.062*	
C5	−0.09168 (16)	0.1453 (2)	−0.08036 (12)	0.0399 (4)	
C6	−0.01715 (15)	0.25332 (19)	−0.01686 (12)	0.0346 (4)	
C7	−0.0495 (2)	0.0586 (2)	−0.15221 (14)	0.0544 (5)	
H7A	−0.1073	−0.0209	−0.1794	0.082*	
H7B	0.0320	0.0181	−0.1190	0.082*	
H7C	−0.0464	0.1232	−0.2041	0.082*	
C8	0.11165 (16)	0.28421 (18)	−0.01751 (12)	0.0347 (4)	
C9	0.32863 (15)	0.31759 (18)	0.09763 (12)	0.0345 (4)	
C10	0.51197 (17)	0.4398 (2)	0.21078 (14)	0.0500 (5)	
H10A	0.5529	0.3457	0.2089	0.060*	
H10B	0.5326	0.4682	0.2792	0.060*	
C11	0.5611 (2)	0.5562 (3)	0.15845 (18)	0.0699 (7)	
H11A	0.5440	0.5266	0.0913	0.105*	
H11B	0.6497	0.5665	0.1905	0.105*	
H11C	0.5211	0.6496	0.1602	0.105*	
C12	0.30146 (18)	0.5213 (2)	0.20352 (13)	0.0428 (4)	
H12A	0.2226	0.5397	0.1514	0.051*	
H12B	0.3449	0.6156	0.2205	0.051*	
C13	0.2756 (2)	0.4621 (3)	0.29216 (14)	0.0573 (6)	
H13A	0.3534	0.4367	0.3436	0.069*	
H13B	0.2332	0.5370	0.3175	0.069*	
H2A	0.187 (2)	0.300 (3)	0.3107 (13)	0.086*	

### Atomic displacement parameters (Å^2^)

**Table:**

	*U*^11^	*U*^22^	*U*^33^	*U*^12^	*U*^13^	*U*^23^
S1	0.0491 (3)	0.0548 (3)	0.0672 (4)	−0.0036 (2)	0.0334 (3)	−0.0109 (3)
O1	0.0528 (8)	0.0629 (9)	0.0344 (7)	−0.0094 (7)	0.0212 (6)	−0.0071 (6)
O2	0.0835 (11)	0.0669 (10)	0.0426 (8)	−0.0108 (8)	0.0366 (8)	0.0027 (7)
N1	0.0331 (7)	0.0443 (9)	0.0310 (7)	−0.0087 (6)	0.0154 (6)	−0.0054 (6)
N2	0.0360 (8)	0.0361 (8)	0.0327 (7)	−0.0062 (6)	0.0075 (6)	0.0006 (6)
C1	0.0438 (10)	0.0458 (11)	0.0438 (10)	−0.0005 (8)	0.0166 (8)	0.0022 (9)
C2	0.0465 (12)	0.0715 (15)	0.0579 (12)	0.0127 (11)	0.0271 (10)	0.0137 (11)
C3	0.0309 (10)	0.0753 (15)	0.0669 (14)	−0.0008 (10)	0.0124 (10)	0.0274 (12)
C4	0.0380 (11)	0.0535 (13)	0.0514 (12)	−0.0092 (9)	0.0000 (9)	0.0140 (10)
C5	0.0364 (9)	0.0389 (10)	0.0362 (9)	−0.0025 (8)	0.0015 (7)	0.0094 (8)
C6	0.0338 (9)	0.0350 (9)	0.0331 (9)	−0.0017 (7)	0.0092 (7)	0.0051 (7)
C7	0.0601 (13)	0.0485 (12)	0.0466 (11)	−0.0125 (10)	0.0078 (10)	−0.0101 (9)
C8	0.0410 (9)	0.0300 (9)	0.0353 (9)	−0.0045 (7)	0.0161 (8)	−0.0052 (7)
C9	0.0372 (9)	0.0346 (9)	0.0337 (9)	−0.0058 (7)	0.0146 (7)	0.0041 (7)
C10	0.0376 (10)	0.0569 (12)	0.0442 (11)	−0.0096 (9)	−0.0007 (8)	0.0021 (9)
C11	0.0515 (13)	0.0740 (16)	0.0800 (16)	−0.0247 (12)	0.0170 (12)	0.0045 (13)
C12	0.0530 (11)	0.0336 (10)	0.0396 (10)	−0.0037 (8)	0.0127 (8)	−0.0036 (8)
C13	0.0747 (15)	0.0627 (14)	0.0372 (10)	0.0000 (11)	0.0227 (10)	−0.0080 (9)

### Geometric parameters (Å, º)

**Table:**

S1—C9	1.6617 (17)	C4—H4	0.9300
O1—C8	1.2176 (19)	C5—C6	1.406 (2)
O2—C13	1.410 (3)	C5—C7	1.503 (3)
O2—H2A	0.815 (10)	C6—C8	1.497 (2)
N1—C8	1.363 (2)	C7—H7A	0.9600
N1—C9	1.411 (2)	C7—H7B	0.9600
N1—H1A	0.8600	C7—H7C	0.9600
N2—C9	1.333 (2)	C10—C11	1.507 (3)
N2—C10	1.465 (2)	C10—H10A	0.9700
N2—C12	1.467 (2)	C10—H10B	0.9700
C1—C2	1.383 (3)	C11—H11A	0.9600
C1—C6	1.388 (2)	C11—H11B	0.9600
C1—H1	0.9300	C11—H11C	0.9600
C2—C3	1.376 (3)	C12—C13	1.507 (3)
C2—H2	0.9300	C12—H12A	0.9700
C3—C4	1.377 (3)	C12—H12B	0.9700
C3—H3	0.9300	C13—H13A	0.9700
C4—C5	1.385 (3)	C13—H13B	0.9700
			
C13—O2—H2A	109.2 (19)	O1—C8—N1	123.46 (15)
C8—N1—C9	127.05 (13)	O1—C8—C6	122.79 (15)
C8—N1—H1A	116.5	N1—C8—C6	113.71 (14)
C9—N1—H1A	116.5	N2—C9—N1	113.03 (14)
C9—N2—C10	120.68 (15)	N2—C9—S1	125.24 (13)
C9—N2—C12	124.08 (14)	N1—C9—S1	121.59 (12)
C10—N2—C12	115.22 (14)	N2—C10—C11	112.60 (16)
C2—C1—C6	121.06 (18)	N2—C10—H10A	109.1
C2—C1—H1	119.5	C11—C10—H10A	109.1
C6—C1—H1	119.5	N2—C10—H10B	109.1
C3—C2—C1	119.02 (19)	C11—C10—H10B	109.1
C3—C2—H2	120.5	H10A—C10—H10B	107.8
C1—C2—H2	120.5	C10—C11—H11A	109.5
C2—C3—C4	120.08 (18)	C10—C11—H11B	109.5
C2—C3—H3	120.0	H11A—C11—H11B	109.5
C4—C3—H3	120.0	C10—C11—H11C	109.5
C3—C4—C5	122.36 (19)	H11A—C11—H11C	109.5
C3—C4—H4	118.8	H11B—C11—H11C	109.5
C5—C4—H4	118.8	N2—C12—C13	113.24 (16)
C4—C5—C6	117.25 (18)	N2—C12—H12A	108.9
C4—C5—C7	119.64 (17)	C13—C12—H12A	108.9
C6—C5—C7	123.09 (16)	N2—C12—H12B	108.9
C1—C6—C5	120.12 (16)	C13—C12—H12B	108.9
C1—C6—C8	119.94 (15)	H12A—C12—H12B	107.7
C5—C6—C8	119.94 (15)	O2—C13—C12	108.07 (15)
C5—C7—H7A	109.5	O2—C13—H13A	110.1
C5—C7—H7B	109.5	C12—C13—H13A	110.1
H7A—C7—H7B	109.5	O2—C13—H13B	110.1
C5—C7—H7C	109.5	C12—C13—H13B	110.1
H7A—C7—H7C	109.5	H13A—C13—H13B	108.4
H7B—C7—H7C	109.5		
			
C6—C1—C2—C3	2.1 (3)	C5—C6—C8—O1	45.3 (2)
C1—C2—C3—C4	−2.0 (3)	C1—C6—C8—N1	43.5 (2)
C2—C3—C4—C5	−0.7 (3)	C5—C6—C8—N1	−136.79 (16)
C3—C4—C5—C6	3.2 (3)	C10—N2—C9—N1	171.38 (14)
C3—C4—C5—C7	−178.29 (18)	C12—N2—C9—N1	−10.0 (2)
C2—C1—C6—C5	0.4 (3)	C10—N2—C9—S1	−4.2 (2)
C2—C1—C6—C8	−179.82 (17)	C12—N2—C9—S1	174.43 (13)
C4—C5—C6—C1	−3.0 (2)	C8—N1—C9—N2	137.07 (16)
C7—C5—C6—C1	178.51 (17)	C8—N1—C9—S1	−47.1 (2)
C4—C5—C6—C8	177.27 (15)	C9—N2—C10—C11	92.3 (2)
C7—C5—C6—C8	−1.2 (3)	C12—N2—C10—C11	−86.5 (2)
C9—N1—C8—O1	−7.0 (3)	C9—N2—C12—C13	92.0 (2)
C9—N1—C8—C6	175.12 (15)	C10—N2—C12—C13	−89.25 (19)
C1—C6—C8—O1	−134.39 (18)	N2—C12—C13—O2	−65.5 (2)

### Hydrogen-bond geometry (Å, º)

**Table:**

*D*—H···*A*	*D*—H	H···*A*	*D*···*A*	*D*—H···*A*
N1—H1*A*···O2	0.86	1.98	2.750 (2)	149
O2—H2*A*···O1^i^	0.81 (2)	1.91 (2)	2.716 (2)	171 (2)

Symmetry code: (i) *x*, −*y*+1/2, *z*+1/2.
